# Joint attention for stimuli on the hands: ownership matters

**DOI:** 10.3389/fpsyg.2015.00543

**Published:** 2015-05-01

**Authors:** J. E. T. Taylor, Jay Pratt, Jessica K. Witt

**Affiliations:** ^1^Department of Psychological Sciences, Purdue University, West Lafayette, IN, USA; ^2^Department of Psychology, University of Toronto, Toronto, ON, Canada; ^3^Department of Psychology, Colorado State University, Fort Collins, CO, USA

**Keywords:** attention, joint action, personal space, embodied cognition, altered vision near the hands

## Abstract

The visual system treats the space near the hands with unique, action-related priorities. For example, attention orients slowly to stimuli on the hands (Taylor and Witt, 2014). In this article, we asked whether jointly attended hands are attended in the same way. Specifically, we examined whether ownership over the hand mattered: do we attend to our hands and the hands of others in the same way? Pairs of participants performed a spatial cueing task with stimuli that could be projected onto one partner’s hands or on a control surface. Results show delayed orienting of attention to targets appearing on the hands, but only for the owner of the hands. For an observer, others’ hands are like any other surface. This result emphasizes the importance of ownership for hand-based effects on vision, and in doing so, is inconsistent with some expectations of the joint action literature.

## Introduction

Imagine shaking someone’s hand. This greeting involves two hands tightly coordinated in a mirrored gesture. To a third party, the two hands appear to act identically: they swoop in open-palmed, grasp their partner, oscillate together, and let go. But for the two people involved in the handshake, the two hands are distinct: their own hand is an effector to be moved and controlled, while the other hand is an object to be acted upon. In social interactions, we often act on or with the hands of others, whether in cooperation (Richardson et al., 2007) or competition (Mori et al., 2002). Consequently, our attention system must regularly gather information about the hands. And although recent research has illuminated how attention treats one’s own hands (Brockmole et al., 2013; Taylor et al., 2015), it remains unknown how attention is allocated to the hands of others. In this paper, we asked whether attention treats one’s own hands and the hands of others differently.

The allocation of attention to stimuli in the space near the hands benefits from a suite of action-related priorities (Brockmole et al., 2013). For example, targets are detected faster in the space near the palms of the hands compared to targets far from the hands, suggesting that attention prioritizes near-hand space (Reed et al., 2006). This effect weakens with distance from the hand, and depends on its orientation, such that the effect disappears when targets appear near the back of the hand (Reed et al., 2010). The hands can also influence how we *ignore* parts of space. Identifying centrally presented targets is normally slowed by the presence of peripheral distractors, when those distractors require a response incompatible with the target (Eriksen and Eriksen, 1974). However, cupping the hands around the target, such that the target appears isolated and the distractors are near the backs of the hands, erases this effect, as though the hands shielded attention from the influence of the distractors (Davoli and Brockmole, 2012). Murchison and Proctor (2015) provided an alternative explanation for this effect, demonstrating that the hands alter attentional processing by providing a flexible frame of reference for directing the focus of attention. Using the same flanker task and postures as Davoli and Brockmole (2012), they altered the task such that the flankers were the targets, and the object cupped within the hands was the distractor. They found a similar reduction in the interfering effect of the distractor when the hands were near the display, suggesting that the hands were not acting as an attentional shield so much as assisting the allocation of attention near them. The attentional prioritization of near-hand space complemented by more thorough processing for stimuli in graspable space. For example, visual search rates are slowed for objects presented near the hands, indicating that attention spends more time on each item in a display (Abrams et al., 2008). Change detection for color is also enhanced in near-hand space (Tseng and Bridgeman, 2011), suggesting that stimuli there benefit from a more detailed representation in visual short-term memory. However, in a similar task, where participants detected changes to color or orientation, the visual system became more sensitive to the action-relevant feature, orientation, than to color (Kelly and Brockmole, 2014). These results support the idea that attention is modulated for stimuli appearing near the hands because of their importance for action.

Another body of evidence supports the idea that hand proximity biases the action-oriented magnocellular processing stream (for a review, see Taylor et al., 2015). This modulated visual pathways (MVP) account of altered vision near the hands describes how stimuli near the hands are processed according to the traits of the magnocellular pathway, such as its transient response and selective tuning for low spatial frequencies (Derrington and Lennie, 1984). In support of the MVP account, stimuli near the hands enjoy better temporal resolution, as evidenced by higher sensitivity on a temporal gap task (Gozli et al., 2012), and weaker masking during object substitution masking (Goodhew et al., 2013). Conversely, the visual system has lower spatial resolution for stimuli near the hands, as evidenced by worse performance on spatial gap detection (Gozli et al., 2012) and a preference for low spatial frequency images (Chan et al., 2013; Abrams and Weidler, 2014). These studies demonstrate how hand position can alter basic, low-level visual processing.

The visual modulation of near-hand space serves to gather and process information for manual actions. This is well established by studies demonstrating a tight coupling between saccades and manual action (Abrams et al., 1990). Eye position during manual action demonstrates a clear tendency for gaze to remain in near-hand space, looking ahead to where the hand will be, and almost never looking back to the hands (Johansson et al., 2001; Land and Hayhoe, 2001). When a tool is used, gaze is displaced ahead of the tool (Land and McLeod, 2000). These patterns of eye movements during manual action suggest that attention is biased to pick up information for what is about to happen; vision for action is all about anticipation.

Consistent with this expectation, we recently demonstrated a cost to orienting attention toward and on the hands (Taylor and Witt, 2014). In these studies, participants performed a spatial cueing task (Posner and Cohen, 1984) where the cue and target locations could appear near or on the hands, such that invalidly cued targets would require orienting attention in the space near the hands, on the hands, or between the hands and the environment. In multiple experiments, we showed that the cost of an invalid cue (i.e., the extra time required to shift attention from the cue to the target) was always greater when orienting attention on the hands (Taylor and Witt, 2014). Given that gaze is typically directed to near-hand space during visually guided manual actions (Land and Hayhoe, 2001), this cost to shifting attention toward or on the hands means that attention will be predisposed to remain entrained to near-hand space, where the targets of actions are most likely to be. By keeping attention in near-hand space, the visual system uses the hands to assist the allocation of attention in the service of future actions.

This same logic applies to hand-eye coordination in social scenarios. One way to establish coordination during joint action is to synchronize attention. Using a similar methodology to the eye-tracking studies reviewed above (Johansson et al., 2001; Land and Hayhoe, 2001), observers of manual actions were shown to employ the same gaze patterns as when they performed the action themselves (Flanagan and Johansson, 2003). That is, when watching another person perform manual actions, the observer’s gaze preceded the actor’s hand, as though they, too, were attending to near-hand space in an anticipatory fashion. If joint actors attend to each other’s hands as they would their own, then the many documented effects of altered vision near and on the hands may also apply to the hands of others.

A common thread through all these studies is that participants viewed only their own hands. Consequently, we cannot be sure whether these hand-related effects on attention depend on ownership of the hand. If these effects exist to serve action, then attention may be specifically tuned to one’s own hands, because only those hands can be controlled. Conversely, attention may be tuned to the sight of hands in general. Such a bias would assist the gathering of information for the actions of others, which could be highly functional in cooperative situations. The question of how we attend to the hands of others is important because a wide range of actions are performed in cooperation with another set of hands.

Such coordination would depend to some extent on joint attention; the ability to direct attention to where a partner is attending. Known as joint attention (Sebanz et al., 2006), in its most basic form it is demonstrated by using eye gaze as a spatial cue for attention: targets are detected faster when preceded by a picture of a face looking in that direction (Friesen and Kingstone, 1998; Frischen et al., 2007). Another reliable cue for joint attention is hand gestures. Hands depicted in a grasping posture cue attention when the target fits the grasp aperture, but inanimate apertures (U-shaped objects) do not (Lindemann et al., 2011). This finding is important for our purposes because it shows that the hands of others can direct attention. But this study was conducted with a single observer watching disembodied hands. The present study examined whether two people, one who owns the hands and one who does not, will direct their attention to those hands in the same way. As we will see, introducing a second person and making it a joint task can change how attention operates.

There are several examples in the literature that show the coordination of perception and action can depend on whether a task is performed jointly or alone (Knoblich et al., 2011). For example, the Simon effect (Simon, 1969) can be distributed across two people when performed together (Sebanz et al., 2003). In this task, participants made left or right responses to the color of a stimulus that pointed left or right. The color was task-relevant, whereas the directionality was irrelevant. Consistent with the classic Simon effect, responses were slow when the response (left or right) and task-irrelevant stimulus dimension are incompatible and fast when they are compatible. In some blocks, subjects performed half of the task, responding to only one color while ignoring the other, effectively making it a go-no go task. Critically, they performed this task either alone or with another person who responded to the other color (a complementary go-no go task). The compatibility effect (faster compatible/slower incompatible responses) emerged only when performing the go-no go tasks together, suggesting that the actors represented each other’s responses (Sebanz et al., 2003; but see Dolk et al., 2013, for an alternative account invoking referential coding instead of shared representation).

Joint attention can also be achieved by attending to a partner’s actions. Attention is slow to process stimuli at recently attended locations, an effect known as inhibition of return (IOR; Posner and Cohen, 1984; Klein, 2000). In a joint IOR task, participants were slower to process stimuli at locations recently reached to by their partners (Welsh et al., 2005, 2007). In this task, participants took turns reaching to targets projected onto a table. When the target appeared at a region recently attended by their partner, they exhibited IOR. Critical to our purposes, this IOR task involved making responses with rapid reaching movements to target locations, and it emerges for different kinds of manual action (Atkinson et al., 2014). Social IOR does not necessarily reflect a co-representation of terminal action goals, but it does show how elements of an actor’s movements can influence their partner’s attention to target locations (Cole et al., 2012). In other words, observers’ attention can be modified by watching their partners perform manual actions.

A similar demonstration of shared representation in joint actions occurs in demonstrations of Fitts’ law. When reaching to targets, movement time (MT) scales to the difficulty of the action (Fitts, 1954). When bimanually tapping between two targets of different difficulties, MTs remain scaled to the harder target, as though compensating to maintain a rhythm (Mottet et al., 2001). Critically, when two people take aim at targets of different difficulties in an alternating, joint tapping task, their MTs scaled to the harder target, even though each participant’s task did not depend on their partner’s target (Fine and Amazeen, 2011). This result shows that participants represented their partner’s task and achieved an interpersonal rhythm that compensated for the harder of the two tasks.

Many of these kinds of joint attention effects are explained by some sort of perspective taking: partners succeed in cooperation because they represent each other’s tasks. Importantly, this co-representation can occur for joint tasks with hand stimuli. Performance on a mental rotation task with hand stimuli (deciding whether two sequentially presented, rotated hands are both left or right hands) depended on whether a partner performed the task concurrently (Böckler et al., 2011). Adopting an allocentric viewpoint (from their partner) allowed participants to complete the task faster than if they maintained their egocentric viewpoint. Thus, there is good reason to believe that hand stimuli may be attended to differently in joint settings compared to alone.

In this study, we asked whether two people in a joint setting would attend to a set of hands the same way. Critically (and perhaps obviously) the hands could not belong to both participants. Stimuli were projected onto the hands of the Owner, while the Observer watched. This variation of the classic spatial cueing task (e.g., Posner et al., 1987) allowed us to assess how attention is allocated to the hands in a social setting. Participants responded to a target that could appear at one of two locations, preceded by a cue. The cue could be presented at the eventual target location (a valid cue), or at the opposite location (an invalid cue). The difference between invalid and valid response times (RTs) reflects the cost to orient attention from one location to the other. When attending to one’s own hands, orienting is very slow (Taylor and Witt, 2014). Our experimental setup allowed us to ask whether people attend to the hands of others in the same way.

We tested two possibilities. First, attention may treat the hands of others like our own. It has been theorized that attention treats the hands differently in order to assist the guidance of action (Taylor and Witt, 2014). Given that we perform so many joint actions, it might be beneficial to attend to the hands of others in the same way, in order to guide attention while watching the hands of others. Indeed, the joint action literature describes numerous situations where performing a task with a partner leads to perspective-taking and a shared representation of action (Sebanz et al., 2006). Thus, Observers and Owners may attend to stimuli on a set of hands in the same way. Specifically, we would expect that attention should orient slower on the hands relative to far from the hands, regardless of whether stimuli appear on one’s own or another’s hands. The other possibility is that ownership matters. Your hands are involved in the execution of action, whereas the hands of others are objects to be acted upon (or with). Consequently, your hands have a different relationship to your visual system than the hands of others. In this case, Owners should display delayed orienting of attention on their hands relative to far from their hands (as in Taylor and Witt, 2014), but Observers should not.

## Materials and Methods

### Participants

Sixty-six students (37 female; six left-handed) from Purdue University participated for course credit.

### Materials and Stimuli

A P2 PicoProjector by AAXA Technologies was mounted 35 cm above a table, projecting downward. Stimuli were a centrally presented cross (1.1 cm × 1.1 cm) flanked by two empty squares (1.0 cm × 1.0 cm; 4.1 cm to the left and right of the cross), and a solid circle (*d* = 0.6 cm). These stimuli were all black.

All participants wore white latex gloves to control for differences in skin tone. In one condition, stimuli were projected onto a pair of objects (roughly 9 cm × 12 cm × 2 cm) made of light brown paper wrapped tightly in white latex. These objects were built to be approximately the same size and thickness of two hands held flat on the table, palms up. We used these objects as a control surface in the condition where stimuli were presented near the hands. Projecting stimuli directly onto the table would have made them appear larger than in the condition where they appear on the hands. In addition, this control surface is made of two distinct objects, like two hands held next to each other. Wrapping them in latex also increased the visual similarity between the hands and the control surface.

### Procedure

**FIGURE 1:**
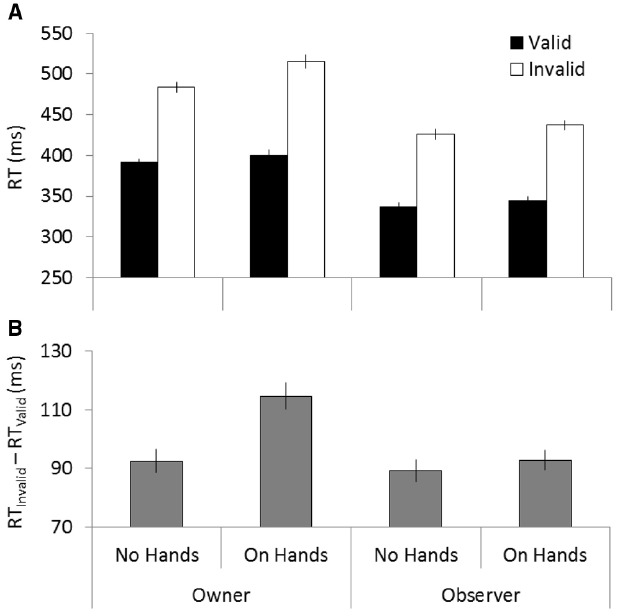
**Time course for a given trial in the On Hands condition.** The No Hands condition is displayed in the top right. The size and contrast of the stimuli have been exaggerated here for visibility.

All participants provided written, informed consent according to the rules and regulations of Purdue University’s institutional review board (IRB). Participants sat across from each other at a small table. Participants were randomly assigned to begin as either Owner or Observer. The Owner held his or her hands in different postures while the Observer simply watched. There were two hand posture conditions. In the On Hands condition, Owners held their hands flat on the table, palms up, and touching so that the two hands formed a contiguous surface—the stimuli were projected down onto their hands. In the No Hands condition, Owners held their hands on their lap, beneath the table—the stimuli were projected down onto two adjacent objects that formed a control surface (see Figure 1).

Each trial began with a central fixation flanked by two squares. After a random delay (1500–3000 ms), the border of one square thickened (+0.2 cm to each side of the square) in order to capture attention. After 200 ms, one of three events occurred: on 70% of trials, the target (a circle) appeared inside the cued square (valid cue); on 20% of trials, the target appeared inside the non-cued square (invalid cue); and on 10% of trials, no target appeared (catch trials). The participants’ task was to identify the location of the target as quickly as possible, and to withhold a response on catch trials. The Owner responded using left and right foot pedals. The Observer responded using left and right mouse buttons. RT was measured from target onset to response. Both participants responded on every trial. Participants were instructed to remain fixated at the central location throughout each trial.

Hand position (On Hands or No Hands) was blocked and randomly ordered, with 60 trials per block. After performing both hand position blocks, the Owner and Observer switched positions and roles. Thus, there were 240 trials in total, for both participants. Cue Validity was balanced at prescribed levels (70% valid, 20% invalid, 10% catch trials).

## Results

Four participants were removed prior to analysis for responding to at least 40% catch trials in either the Owner or Observer conditions. Another five participants were removed because they responded with exceptionally poor accuracy (>3 SDs from the mean). Among remaining participants, incorrect responses were rare (<5% of responses to non-catch trials) and were also removed from analysis. RTs faster than 100 ms and slower than 1000 ms were excluded to obscure errors of apprehension and lapses of attention (these are the same RT exclusion criteria used in Reed et al., 2006; Taylor and Witt, 2014). These trials comprised 2.0% of correct responses to non-catch trials.^1^

**FIGURE 2:**
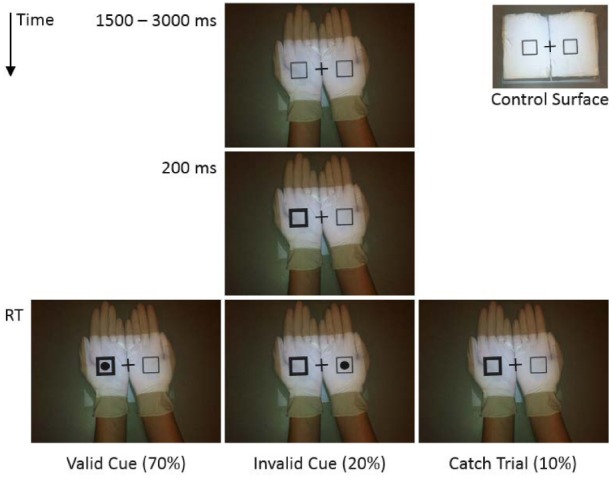
**Response time plotted as a function of cue validity, hand position, and role (A).** Participants were slower to respond to invalidly cued targets on their hands relative to off their hands, but only when stimuli appeared on their own hands and not when they appeared on another person’s hands. The cost to detecting an invalidly cued target is expressed as the difference between RTs for validly and invalidly cued targets **(B)**. Error bars represent one within-subjects standard error of the mean.

To assess how orienting attention on one’s own hands compared to orienting attention on another person’s hands, we conducted a 2 (Role: Owner vs. Observer) × 2 (Hand Position: On Hands vs. No Hands) × 2 (Cue Validity: Valid Cue vs. Invalid Cue) repeated-measures ANOVA. Confirming the basic result of the spatial cueing paradigm, validly cued targets were detected faster than invalidly cued targets, indicating a main effect of Cue Validity, *F*(1,56) = 1138.62, *p* < 0.001, ${\text{η}}_{p}^{2}$ = 0.95. In addition, the position of the hands influenced RTs, indicating a main effect of Hand Position, *F*(1,56) = 8.96, *p* = 0.004, ${\text{η}}_{p}^{2}$ = 0.14. The participants’ current role also influenced RTs, as Observers were faster to respond than Owners, indicating a main effect of Role, *F*(1,56) = 39.73, *p* < 0.001, ${\text{η}}_{p}^{2}$ = 0.41. Critically, the effect of delayed orienting on the hands depended on the participants’ current role. There was also a three-way interaction between Role, Cue Validity, and Hand Position, *F*(1,56) = 4.42, *p* = 0.04, ${\text{η}}_{p}^{2}$ = 0.07 (see Figure 2).

A *post hoc* analysis comparing the cueing effects (difference in RT for valid and invalid cues) for all four conditions (Owner On Hands, Owner No Hands, Observer On Hands, Observer No Hands) revealed that the cost of orienting attention was greater when Owner responded to stimuli on their own hands compared to any other condition, all *t*s > 3.07, all *p*s < 0.003. No other comparison reached significance, all *t*s < 0.64, all *p*s > 0.525.

### Owner Data

To assess how participants attended to stimuli presented on their own hands, we ran a 2 (Hand Position: On Hands vs. No Hands) × 2 (Cue Validity: Valid Cue vs. Invalid Cue) repeated-measures ANOVA with RTs from the Owner. Validly cued targets were detected faster than invalidly cued targets, indicating a main effect of Cue Validity, *F*(1,56) = 620.50, *p* < 0.001, ${\text{η}}_{p}^{2}$ = 0.92. In addition, the position of the hands influenced RTs, indicated by a main effect of Hand Position, *F*(1,56) = 7.46, *p* = 0.008, ${\text{η}}_{p}^{2}$ = 0.12. Owners were slower to detect targets on their hands compared to off the hands. Critically, the strength of the cueing effect depended on the position of the hands; the interaction between Cue Validity and Hand Position, *F*(1,56) = 9.45, *p* = 0.003, ${\text{η}}_{p}^{2}$ = 0.14 was significant. A *post hoc* analysis comparing the cueing effect for the On Hands and No Hands conditions reveal that participants were especially slow to respond to targets appearing on their own hands relative to when their hands were far from the display, *t*(56) = 3.07, *p* = 0.003, *d* = 0.58.

### Observer Data

To assess whether attention orients slowly on someone else’s hands, we conducted the same analyses described for the Owners with RTs from the Observers. Validly cued targets were detected faster than invalidly cued targets, indicating a main effect of Cue Validity, *F*(1,56) = 910.50, *p* < 0.001, ${\text{η}}_{p}^{2}$ = 0.94. In addition, the position of the hands influenced RTs, indicated by a main effect of Hand Position, *F*(1,56) = 4.33, *p* = 0.042, ${\text{η}}_{p}^{2}$ = 0.07, with Observers slower to detect targets on the Owners’ hands compared to off the hands. Critically, the strength of the cueing effect was independent of the position of the hands, as there was no interaction between Cue Validity and Hand Position, *F*(1,56) = 0.41, *p* = 0.525, ${\text{η}}_{p}^{2}$ = 0.01. A *post hoc* analysis comparing the validity effect for the On Hands and No Hands conditions confirms that there was no difference between the cost to shifting attention on someone else’s hands versus when there were no hands near the display, *t*(56) = 0.64, *p* = 0.525, *d* = 0.12.

## Discussion

This experiment was designed to test how a single set of hands is attended by two people. There were two possible, competing outcomes. One outcome would be that the hand-based effect on attention would transfer to an Observer when a set of hands were jointly attended. This possibility, based on studies showing how joint action and joint attention can evoke a shared representation between two people, predicts that attention should treat jointly attended hands the same, regardless of who they belong to. In this case, we predicted that both Owner and Observer would show delayed orienting of attention to stimuli on the hands (as in Taylor and Witt, 2014). Alternatively, it may be that ownership of the hand is necessary for the hand-based effect on attention. There are approximately 14 billion hands on the planet, and they are all visually similar. One’s own two hands, however, can be willed to action and are therefore unique. Consequently, in terms of the present study, only the Owners might display delayed orienting of attention for stimuli on the hands. The results of this study support the second prediction. Orienting attention to stimuli on the hands is slow compared to orienting attention to stimuli that appear far from the hands, but only when appearing on one’s own hands.

The results from the present experiment replicates the original effect described by Taylor and Witt (2014), where attention oriented more slowly to, from, or on the hands compared to near or far from the hands. Importantly, the present results also imply that the effect of slow attentional orienting on the hands depends on ownership of the hands. Simply put, it is not enough for stimuli to appear *on* hands. Those hands must be controllable. This caveat is reminiscent of recent work demonstrating the importance of action intentions on action-related perceptual biases. For example, stimuli beyond reach are perceived as closer when holding a tool that brings them within reach, but only when the perceiver intends to use the tool (Witt et al., 2005). In our study, the Owner is capable of using their hands, and intends to react to stimuli presented on them (although not with them), whereas the Observer cannot act with those hands—they are just objects in the world. However, it remains unclear when these effects of action on perception occur in observers (e.g., Bloesch et al., 2012; Witt et al., 2012, 2014), and when they do not.

It bears mention that the present research used a localization task, whereas the Taylor and Witt (2014) paper used a simple detection task (participants pressed a button as soon as a target appeared, regardless of location). In both cases, however, attention must shift across locations, and the comparisons between results are permissible. Indeed, localization should be more attentionally effortful than a simple detection (Treisman and Gelade, 1980). Thus, the present results also show how delayed orienting of attention replicates for a localization task. It should also be noted that the main effect of role (Owner vs. Observer) was likely due to the effectors involved in responding. The foot pedal responses were significantly longer than the mouse responses. This may present some concern that the critical effect of longer orienting on one’s own hands is merely hidden by the shorter responses in the Observer condition. However, we have demonstrated this effect in the past using both mouse responses and foot pedals (Taylor and Witt, 2014, Experiments 1A and 1B, respectively). It is reasonable to expect that if the effect were there, Observers would have shown it with mouse responses (in fact, the mouse responses collected in our past study were faster than here, suggesting any effect in the Observer condition should have been detectable).

Interestingly, the cost of orienting attention was the same when the Owner viewed stimuli far from their hands, and when the Observer viewed stimuli either on or far from the Owner’s hands (see Figure 2B). This result suggests that the Observer attended to stimuli on the Owner’s hands as though it were any other surface. Attentionally speaking, another person’s hand is simply an object to be acted on. This interpretation equates the hands of others with, more or less, other objects in the environment. Therefore, the delayed orienting of attention on the hands described by Taylor and Witt (2014) was not simply triggered by the sight of hands. Rather, some autonomous control over the hand seems necessary to elicit the effect.

Alternatively, it is possible that ownership over the hands increased the degree to which the hands were perceptually separable as distinct objects, and that this stronger perceptual separability caused orienting between objects to be even slower. The effect of delayed orienting between objects versus within a single object is a well-documented expression of object-based attention (Egly et al., 1994). If ownership increases that perceptual separability of the hands, it may explain why we observed the delayed orienting on the hands for the Owners but not the Observers. However, we explicitly tested the possibility that delayed orienting on the hands is an expression of object-based attention in an earlier study (Experiment 3, Taylor and Witt, 2014). We found delayed orienting of attention on the hands regardless of whether cue and target were presented at different locations on a single hand or across both hands relative to the control surfaces. This suggests the delayed orienting due to the hand is not akin to Egly et al. (1994) classic demonstration of object-based attention. It therefore seems unlikely that ownership affects delayed orienting on the hands via some expression of object-based attention.

That one’s own hands are attentionally privileged whereas others are *just* objects implies that the visual system is tuned to ownership. And yet, the literature on joint action suggests that there should be some measure of shared representation between coordinated actors. The hands of others can guide attention (Lindemann et al., 2011), and jointly attending to hands invokes a shared representation (Welsh et al., 2005; Böckler et al., 2011), so we expected that jointly attending to a set of hands might evoke similar representations between individuals. Indeed, the joint action literature is full of cases where performing actions with a partner causes a distribution of cognitive processes across the participants (Sebanz et al., 2006; Knoblich et al., 2011). Instead, we found that only the Owner displayed effects of hand-based attention. In this case, the primacy of ownership overruled the co-representation of joint attention. Thus, the current results stand out as an exception to typical demonstrations of shared representations during joint action.

Although the present study contradicts the expectation that observers should co-represent the task with their partners, it is worth noting an important difference between our method and those typically employed in the joint action literature. Those experiments typically involve distributing task demands across participants, such that performance depends on a shared action representation (Sebanz et al., 2006). For example, in the joint Simon task, each participant only responds to one half of the stimuli, while withholding responses to the other stimuli (Sebanz et al., 2003). In the present study, the task was not divided: both participants represented the entire action. This may have disincentivized shared representations or perspective-taking, and as such our results do not necessarily contradict the joint action literature. Notwithstanding, the expectation that observers should be able to represent another person’s action—and thereby demonstrate similar effects on attention and perception—is established in the literature (Samson et al., 2010; Bloesch et al., 2012; Witt et al., 2012).

In conclusion, the evidence supports the idea that hand-based effects on attention are restricted by ownership. Attention is different for stimuli appearing on the hands, but only for one’s own hands. We originally proposed that this effect serves to assist action by biasing attention away from the hands and toward near-hand space, where the targets of action are typically located (Taylor and Witt, 2014). The evidence presented here suggests the hands of others cannot be used for the same advantage. Reconsider the handshake example presented at the beginning of this article. In light of new evidence, attention does not treat your partner’s hand as a twin. It is the target of your action, and nothing more.

### Conflict of Interest Statement

The authors declare that the research was conducted in the absence of any commercial or financial relationships that could be construed as a potential conflict of interest.

## Footnotes

1 A reviewer raised the point that arbitrary RT cutoffs (e.g., 100–1000 ms) would exclude more trials in the Owner than Observer conditions, because Owner responses were longer. To address this, we conducted an additional analysis by calculating the mean RT and SD for the Owner and Observer responses, and excluding trials that were <100 ms or >3 SDs for those means. This method would ensure that an equal number of trials were excluded in the Owner and Observer conditions. The results were similar to those reported in the main text. The critical three-way interaction remained significant, as well as the interaction between hand position and cue validity for Owners, but not for Observers. The cueing effect for the Owner, On Hands condition remained longer than all other conditions. The means and SDs (in parentheses) follow. For the Owner condition: On Hands, Valid—393.88 (82.01); On Hands, Invalid—500.40 (84.98); No Hands, Valid—388.16 (64.80); No Hands, Invalid—479.29 (72.48). For the Observer condition: On Hands, Valid—334.90 (40.85); On Hands, Invalid—416.40 (37.88); No Hands, Valid—327.79 (38.49); No Hands, Invalid—411.59 (39.66).

## References

[B1] Abrams R. A. Davoli C. C. Du F. Knapp W. H. III Paull D. (2008). Altered vision near the hands. Cognition 107, 1035–1047. 10.1016/j.cognition.2007.09.006 17977524

[B2] Abrams R. A. Meyer D. E. Kornblum S. (1990). Eye-hand coordination: oculomotor control in rapid aimed limb movements. J. Exp. Psychol. Hum. Percept. Perform. 16, 248. 10.1037/0096-1523.16.2.248 2142197

[B3] Abrams R. A. Weidler B. J. (2014). Trade-offs in visual processing for stimuli near the hands. Atten. Percept. Psychophys. 76, 383–390. 10.3758/s13414-013-0583-1 24222266

[B4] Atkinson M. A. Simpson A. Skarratt P. A. Cole G. G. (2014). Is social inhibition of return due to action co-representation? Acta Psychol. 150, 85–93. 10.1016/j.actpsy.2014.04.003 24859672

[B5] Bloesch E. K. Davoli C. C. Roth N. Brockmole J. R. Abrams R. A. (2012). Watch this! Observed tool use affects perceived distance. Psychon. Bull. Rev. 19, 177–183. 10.3758/s13423-011-0200-z 22231725

[B6] Böckler A. Knoblich G. Sebanz N. (2011). Giving a helping hand: effects of joint attention on mental rotation of body parts. Exp. Brain Res. 211, 531–545. 10.1007/s00221-011-2625-z 21455620 PMC3102195

[B7] Brockmole J. R. Davoli C. C. Abrams R. A. Witt J. K. (2013). The world within reach: effects of hand posture and tool use on cognition. Curr. Dir. Psychol. Res. 22, 38–44 10.1177/0963721412465065

[B8] Chan D. Peterson M. A. Barense M. D. Pratt J. (2013). How action influences object perception. Front. Psychol. 4:462. 10.3389/fpsyg.2013.00462 23885247 PMC3717510

[B9] Cole G. G. Skarratt P. A. Billing R. C. (2012). Do action goals mediate social inhibition of return? Psychol. Res. 76, 736–746. 10.1007/s00426-011-0395-7 22143901

[B10] Davoli C. C. Brockmole J. R. (2012). The hands shield attention from visual interference. Atten. Percept. Psychophys. 74, 1386–1390. 10.3758/s13414-012-0351-7 22855428

[B11] Derrington A. M. Lennie P. (1984). Spatial and temporal contrast sensitivities of neurones in lateral geniculate nucleus of macaque. J. Physiol. 357, 219–240. 10.1113/jphysiol.1984.sp015498 6512690 PMC1193256

[B12] Dolk T. Hommel B. Prinz W. Liepelt R. (2013). The (not so) social Simon effect: a referential coding account. J. Exp. Psychol. Hum. Percept. Perform. 39, 1248. 10.1037/a0031031 23339346

[B13] Egly R. Driver J. Rafal R. D. (1994). Shifting visual attention between objects and locations: evidence from normal and parietal lesion subjects. J. Exp. Psychol. Gen. 123, 161. 10.1037/0096-3445.123.2.161 8014611

[B14] Eriksen B. A. Eriksen C. W. (1974). Effects of noise letters upon identification of a target letter in a non-search task. Percept. Psychophys. 16, 143–149 10.3758/BF03203267

[B15] Fine J. M. Amazeen E. L. (2011). Interpersonal Fitts’ law: when two perform as one. Exp. Brain Res. 211, 459–469. 10.1007/s00221-011-2707-y 21547558

[B16] Fitts P. (1954). The information capacity of the human motor system in controlling the amplitude of movement. J. Exp. Psychol. 47, 381–391. 10.1037/h0055392 13174710

[B17] Flanagan J. R. Johansson R. S. (2003). Action plans used in action observation. Nature 424, 769–771. 10.1038/nature01861 12917683

[B18] Friesen C. K. Kingstone A. (1998). The eyes have it! Reflexive orienting is triggered by nonpredictive gaze. Psychon. Bull. Rev. 5, 490–495 10.3758/BF03208827

[B19] Frischen A. Bayliss A. P. Tipper S. P. (2007). Gaze cueing of attention: visual attention, social cognition, and individual differences. Psychol. Bull. 133, 694. 10.1037/0033-2909.133.4.694 17592962 PMC1950440

[B20] Goodhew S. C. Gozli D. G. Ferber S. Pratt J. (2013). Reduced temporal fusion in near-hand space. Psychol. Sci. 24, 891–900. 10.1177/0956797612463402 23599307

[B21] Gozli D. G. West G. L. Pratt J. (2012). Hand position alters vision by biasing processing through different visual pathways. Cognition 124, 244–250. 10.1016/j.cognition.2012.04.008 22633129

[B22] Johansson R. S. Westling G. Backstrom A. Flanagan J. R. (2001). Eye-hand coordination in object manipulation. J. Neurosci. 21, 6917–6932. 11517279 10.1523/JNEUROSCI.21-17-06917.2001 PMC6763066

[B23] Kelly S. P. Brockmole J. R. (2014). Hand proximity differentially affects visual working memory for color and orientation in a binding task. Front. Psychol. 5:318. 10.3389/fpsyg.2014.00318 24795671 PMC4001000

[B24] Klein R. M. (2000). Inhibition of return. Trends Cogn. Sci. 4, 138–147 10.1016/S1364-6613(00)01452-2 10740278

[B25] Knoblich G. Butterfill S. Sebanz N. (2011). “Psychological research on joint action: theory and data,” in Psychology of Learning and Motivation: Advances in Research and Theory, Vol. 54, ed. Ross B. (Burlington: Academic Press), 59.

[B26] Land M. F. Hayhoe M. (2001). In what ways do eye movements contribute to everyday activities? Vision Res. 41, 3559–3565. 10.1016/S0042-6989(01)00102-X 11718795

[B27] Land M. F. McLeod P. (2000). From eye movements to actions: how batsmen hit the ball. Nat. Neurosci. 3, 1340–1345. 10.1038/81887 11100157

[B28] Lindemann O. Nuku P. Rueschemeyer S. A. Bekkering H. (2011). Grasping the other’s attention: the role of animacy in action cueing of joint attention. Vision Res. 51, 940–944. 10.1016/j.visres.2010.12.009 21215275

[B29] Mori S. Ohtani Y. Imanaka K. (2002). Reaction times and anticipatory skills of karate athletes. Hum. Mov. Sci. 21, 213–230. 10.1016/S0167-9457(02)00103-3 12167300

[B30] Mottet D. Guiard Y. Ferrand T. Bootsma R. J. (2001). Two-handed performance of a rhythmical fitts task by individuals and dyads. J. Exp. Psychol. Hum. Percept. Perform. 27, 1275. 10.1037/0096-1523.27.6.1275 11766924

[B31] Murchison N. M. Proctor R. W. (2015). How hand placement modulates interference from extraneous stimuli. Atten. Percept. Psychophys. 34, 0–352. 10.3758/s13414-014-0765-5 25239096

[B32] Posner M. I. Cohen Y. (1984). “Components of visual orienting,” in Attention and Performance X: Control of Language Processes, Vol. 32, eds Bouma H. Bouwhuis D. G. (Hillsdale, NJ: Erlbaum), 531–556.

[B33] Posner M. I. Walker J. A. Friedrich F. J. Rafal R. D. (1987). How do the parietal lobes direct covert attention? Neuropsychologia 25, 135–146. 10.1016/0028-3932(87)90049-2 3574646

[B34] Reed C. L. Betz R. Garza J. P. Roberts R. J. Jr. (2010). Grab it! Biased attention in functional hand and tool space. Atten. Percept. Psychophys. 72, 236–245. 10.3758/APP.72.1.236 20045892

[B35] Reed C. L. Grubb J. D. Steele C. (2006). Hands up: attentional prioritization of space near the hand. J. Exp. Psychol. Hum. Perform. Percept. 32, 166–177. 10.1037/0096-1523.32.1.166 16478334

[B36] Richardson M. J. Marsh K. L. Baron R. M. (2007). Judging and actualizing intrapersonal and interpersonal affordances. J. Exp. Psychol. Hum. Percept. Perform. 33, 845. 10.1037/0096-1523.33.4.845 17683232

[B37] Samson D. Apperly I. A. Braithwaite J. J. Andrews B. J. Bodley Scott S. E. (2010). Seeing it their way: evidence for rapid and involuntary computation of what other people see. J. Exp. Psychol. Hum. Percept. Perform. 36, 1255–1266. 10.1037/a0018729 20731512

[B38] Sebanz N. Bekkering H. Knoblich G. (2006). Joint action: bodies and minds moving together. Trends Cogn. Sci. 10, 70–76. 10.1016/j.tics.2005.12.009 16406326

[B39] Sebanz N. Knoblich G. Prinz W. (2003). Representing others’ actions: just like one’s own? Cognition 88, 11–21. 10.1016/S0010-0277(03)00043-X 12804818

[B40] Simon J. R. (1969). Reactions towards the source of stimulation. J. Exp. Psychol. 81, 174–176. 10.1037/h0027448 5812172

[B41] Taylor J. E. T. Gozli D. G. Chan D. Huffman G. Pratt J. (2015). A touchy subject: advancing the modulated visual pathways account of altered vision near the hands. Transl. Neurosci. 6, 1–7. 10.1515/tnsci-2015-0001 PMC4936609 28123785

[B42] Taylor J. E. T. Witt J. K. (2014). Altered attention for stimuli on the hands. Cognition 133, 211–225. 10.1016/j.cognition.2014.06.019 25051509

[B43] Treisman A. M. Gelade G. (1980). A feature-integration theory of attention. Cogn. Psychol. 12, 97–136 10.1016/0010-0285(80)90005-5 7351125

[B44] Tseng P. Bridgeman B. (2011). Improved change detection with nearby hands. Exp. Brain Res. 209, 257–269. 10.1007/s00221-011-2544-z 21279633 PMC3041905

[B45] Welsh T. N. Elliott D. Anson J. G. Dhillon V. Weeks D. J. Lyons J. L. (2005). Does Joe influence Fred’s action? Inhibition of return across different nervous systems. Neurosci. Lett. 385, 99–104. 10.1016/j.neulet.2005.05.013 15927370

[B46] Welsh T. N. Lyons J. Weeks D. J. Anson J. G. Chua R. Mendoza J. (2007). Within-and between-nervous-system inhibition of return: observation is as good as performance. Psychon. Bull. Rev. 14, 950–956. 10.3758/BF03194127 18087965

[B47] Witt J. K. Proffitt D. R. Epstein W. (2005). Tool use affects perceived distance, but only when you intend to use it. J. Exp. Psychol. Hum. Percept. Perform. 31, 880–888. 10.1037/0096-1523.31.5.880 16262485

[B48] Witt J. K. South S. C. Sugovic M. (2014). A perceiver’s own abilities influence perception, even when observing others. Psychon. Bull. Rev. 21, 384–389. 10.3758/s13423-013-0505-1 24002969

[B49] Witt J. K. Sugovic M. Taylor J. E. T. (2012). Action-specific effects in a social context: others’ abilities influence perceived speed. J. Exp. Psychol. Hum. Percept. Perform. 38, 715. 10.1037/a0026261 22103758

